# Prevalence of Cardiovascular Disease Risk Factors in the Women Population Covered by Health Centers in Ardabil

**DOI:** 10.1155/2022/2843249

**Published:** 2022-02-27

**Authors:** Leli Avesta, Sina Rasoolzadeh, Mahdi Naeim, Aziz Kamran

**Affiliations:** ^1^Department of Cardiology, Faculty of Medicine, Ardabil University of Medical Sciences, Ardabil, Iran; ^2^School of Medicine and Allied Medical Sciences, Social Determinants of Health Research Center, Ardabil University of Medical Sciences, Ardabil, Iran; ^3^Social Determinants of Health Research Center, Ardabil University of Medical Sciences, Ardabil, Iran

## Abstract

**Objective:**

This study aimed to determine the prevalence of cardiovascular risk factors in the population of women aged 30 to 60 years covered by health centers in Ardabil.

**Methods:**

This retrospective descriptive-analytical study was conducted on 1006 women aged 30 to 60 years who were covered by Ardabil comprehensive urban health service centers, and they were selected by using the multistage random sampling method. In the first stage, health centers in Ardabil were divided into five geographical areas, and the population covered by each of the five areas was calculated. In the second stage, the number of samples was allocated as a quota in the regions, and in the third stage, in proportion to the population covered by each center in Ardabil, the samples were selected. Women with one of the conditions of pregnancy, lactation, history of kidney disease, known diabetes under medication, history of hepatitis, history of cardiovascular surgery, and history of cancer were excluded from the research process.

**Results:**

The average activity of individuals was 24.42 minutes per day. The mean intake of fruits and vegetables was 1.9 ± 0.9 and 2.1 ± 1.07 unit/day, respectively, and meat was 286.6 ± 174.6 grams per week. The mean of HDL, LDL, TG, cholesterol, and FBS were 43.6 ± 10.4, 101.28 ± 26.3, 159.89 ± 54.01, 185.99 ± 37.9, and 94.62 ± 13.3 mg/dl, respectively. The mean systolic blood pressure (SBP) and diastolic blood pressure (DBP) were 108.14 and 68.26 mmHg, respectively.

**Conclusions:**

Abdominal obesity (waist above 88), obesity and overweight (high body mass index), high triglycerides, high cholesterol, and LDL and HDL outside the proper range were the most important and risk factors for cardiovascular disease among women.

## 1. Introduction

Cardiovascular diseases are one of the most important health problems and one of the most growing diseases. Today, these diseases are associated with a very high prevalence in both developed and developing countries and are considered one of the most important causes of death [1]. According to the World Health Organization, about 30 percent of all deaths are due to cardiovascular disease each year [2]. According to studies conducted in Iran, the highest risk of cardiovascular disease in the population is associated with high blood pressure [3].

The prevalence of hypertension in the adult population over 18 years is between 17.3% and more than 20% [4], and about 26.9% in the population is between 25 and 64 years in Iran [5]. The present study examines the prevalence of cardiovascular risk factors in the female population of Ardabil. Identification of these risk factors can be effective in predicting the likelihood of advanced cardiovascular disease and its complications. Knowing the regional prevalence of these risk factors, appropriate measures to prevent the progression of these diseases, and early treatment can be put on the agenda. There are many risk factors for heart disease: age, sex, smoking, physical inactivity, excessive alcohol consumption, unhealthy diet, obesity, genetic predisposition and family history of cardiovascular disease, hypertension (hypertension), hyperglycemia (diabetes mellitus), high blood cholesterol (hyperlipidemia), undiagnosed celiac disease, psychosocial factors, poor economic status and poverty, low level of education, and air pollution [6].

Some of these risk factors, such as age, gender, or family history/genetic predisposition, are immutable. However, many important cardiovascular factors can be corrected by lifestyle changes, social changes, and medication (e.g., prevention of hypertension, hyperlipidemia, and diabetes). People with obesity are at risk for coronary heart disease [7].

Many studies have been conducted over the past years on the prevalence of risk factors for noncommunicable diseases, including cardiovascular disease in Iran and its cities and in other parts of the world. In an honest business study conducted in 2006 in Ardabil province, 29% of men and 2.6% of women consumed tobacco products daily and the average body mass index of individuals was reported to be 26.6 kg/m^2^. The incidence and increase of obesity have been directly related to high blood pressure in the individuals. Fruit consumption per day was reported to be 1.1 per person, and 33% had a sedentary lifestyle [8]. Behzad et al. conducted a study in 2015 on 460 heart patients in Babol city who underwent coronary artery bypass graft surgery and found a significant prevalence of blood pressure, diabetes, and obesity and the prevalence of the risk factors was high in women [9]. In a similar study conducted in Tabriz in 2017, the prevalence of general obesity and abdominal obesity was significant. There was a very significant relationship between oxidized LDL (serum oxidized LDL) and aging and also between serum hs-CRP and DBP and triglyceride levels [10]. In the study of Moghimi-Dehkordi et al., overweight and obesity were reported to be 36.9% and 20.6% in 2008 in Tehran women, respectively [11].

In general, in similar studies, factors such as smoking, obesity, blood pressure, dietary patterns and mobility, income and economic factors, family history, and laboratory cases such as triglycerides, cholesterol, sodium, vitamin D, and zinc were considered risk factors of cardiovascular disease and were investigated. In the present study, an attempt was made to examine demographic and behavioral variables along with clinical and laboratory cases in a wide range of women in Ardabil.

## 2. Method

This descriptive-analytical study was conducted on 1006 women aged 30 to 60 years who were under the auspices of Ardabil comprehensive urban health service centers, and they were selected by using the multistage sampling method. In the first stage, Ardabil health centers are divided into five geographical areas (Region 1: city center; Region 2: South; Region 3: West; Region 4: North; and Region 5: East) and the population covered by each region was calculated. In the second stage, the number of samples was allocated as a quota in the allocated areas, and in the third stage, by referring to all health centers in Ardabil, in proportion to the population covered by each center, the population was sampled from the share of the allocated area. Finally, 224 persons (22.3%) from Region 1, 208 (20.7%) from Region 2, 147 (14.6%) from Region 3, 205 (20.4%) from Region 4, and 222 (22.1%) from Region 5 participated in the present study.

By referring to the databases of comprehensive urban health service centers and using the information systems of the desired databases, demographic information of the referring people during 2019 and 2020 with a history of at least ten years of residence in the area, including age, level of education, marital status, and occupational status; the latest anthropometric data recorded including weight, height, waist, and calculation of body mass index; daily consumption of vegetables and fruits; daily activities of the people; smoking; using other drugs; and the latest test results of the patients, such as fasting blood sugar, cholesterol levels, HDL and LDL, and triglycerides, were collected and evaluated. For all the subjects, three blood pressures recorded in the electronic record during the past year were collected and the mean systole and diastole of these three blood pressures were calculated. According to the latest national protocol for monitoring blood pressure provided by the Office of Non-Communicable Diseases Management of the Ministry of Health and Medical Education, SBP less than 120 and DBP less than 80 mmHg were considered normal blood pressure, SBP between 120 and 139 and DBP between 80 and 90 mmHg were considered prehypertension, and SBP above 140 and DBP above 90 mmHg were considered hypertension. Body mass index (BMI), which describes the status of general obesity, is calculated as weight in kilograms divided by height squared in meters. BMI groups were defined and categorized as low weight (BMI <18.5 kg/m^2^), healthy and normal weight (BMI = 18.5–24.9 kg/m^2^), overweight (BMI = 25.0–29.9 kg/m^2^), and obesity (BMI> 30 kg/m^2^). Abdominal obesity was considered as waist circumference equal to or greater than 88 cm. In clinical trials, the triglyceride level less than 150 (TG < 150) was considered normal, between 150 and 199 (TG = 150–199) borderline, between 200 and 499 (TG = 200–499) high, and more than 500 (TG > 500) was considered too high. Cholesterol less than 200, HDL equal to and above 50, and LDL less than 130 were defined as optimal and normal values [12, 13].

Women with a history of pregnancy, lactation, kidney disease, known diabetes under medical treatment, hepatitis, cardiovascular surgery, and cancer and people with incomplete electronic file information were excluded from the study.

To comply with the principles of medical ethics, after obtaining the code of ethics (IR.ARUMS.REC.1399.429) by the Ethics Committee of Ardabil University of Medical Sciences and presenting it to health centers, information of individuals without access to identity information, kept confidential, and the results will be reported without mentioning the name.

Data were collected using SPSS software, version 22, and analyzed using independent statistical tests, one-way analysis of variance, multiple linear regression, and equivalent nonparametric tests with a significance level of less than 5%.

## 3. Results

The majority of participants were married (967, 96.1%), had high waist size (590, 58.6%), and were overweight (431, 42.8%).  Table 1 shows the frequency distribution of demographic variables in the study.

Table 2 shows the frequency distribution of clinical laboratory variables of the study. In the present study, the majority of participants had normal SBP (930, 92.4%), normal DBP (957, 95.1%), borderline TG (389, 38.7%), normal Chol (682, 67.8%), low HDL (777, 77.2%), and normal LDL (861, 85.6%).

The mean of age, height, weight, BMI, and waist circumstance were 42.9 ± 8.4 years, 161.5 ± 6.7 cm, 74.2 ± 11.3 kg, 28.5 ± 5.09 kg/m^2^, and 91.3 ± 10.4 cm, respectively. The mean intake of fruits and vegetables was 1.9 ± 0.9 and 2.1 ± 1.07 unit/day, respectively, and meat was 286.6 ± 174.6 (Table 3).

The results showed that there were significant differences in the mean of HDL, LDL, TG, FBS, Chol, SBP, and DBP between the normal and obese participants (Table 4).

The results showed that the linear regression model predicted 41.8% of changes in SBP (Table 5).

The results showed that the linear regression model predicted 39.6% of changes in DBP (Table 6).

## 4. Discussion and Conclusion

In terms of body mass index, 431 people (42.8%) were overweight, which included more frequency. 35.1% were in the obese group. The average waist size was 91.22 cm, and based on the waist size, 58.6% had abdominal obesity. According to the results, overweight and general obesity and abdominal obesity are very common in our study population. In the study of Savadpour et al., which was performed on 354 people over 30 years old in Ardabil in 2014, the prevalence of overweight and obesity was reported to be 18.1% and 32.5%, respectively [14]. In the study of Moghimi-Dehkordi et al., overweight and obesity were reported to be 36.9% and 20.6% in 2008 in Tehran women, respectively [11]. In the study of Hajian-Tilaki and Heidari on the female population of northern Iran, overweight, obesity, and abdominal obesity were reported to be 34.8%, 18.8%, and 28.3%, respectively [15].

The rate of obesity was higher in our study than these three studies, which indicates a higher rate of obesity and overweight in Ardabil women than women in Tehran and northern Iran, which is necessary to find the cause and plan to remove obstacles in this area. In the study of Heshmat et al. on 5724 adults in five metropolises of Iran, the prevalence of overweight and obesity was reported to be 38.5% and 19.7%, respectively. Abdominal obesity was reported to be 45.1% in women and 19.6% in men based on waist circumference. In this study, the cut-off point for waist size in people with a BMI greater than 30, for men 99.5 cm and women 94.25 cm, is presented [16], which is 88 cm in our study. The prevalence of overweight and general and abdominal obesity is lower in our study.

Our results on obesity rates were even higher than those reported for women in some other countries. In the study by Reynolds et al. of Chinese women, the rates of overweight and obesity were 26.1% and 5%, respectively [17]. According to the National Health and Nutrition Survey (NHANES), the prevalence of general obesity in US women is 34% [18]. In a 2006 study by Bahrami et al., the prevalence of overweight and obesity in the Iranian population was 62.2% and 28%, respectively, and showed that obesity, overweight, and hypertension were as prevalent in Iran as in the United States. But Iranian women are fatter than American women, and it shows that health policymakers need to address this gender gap as a major issue [19].

Regular consumption of fruits and vegetables in the diet has always been one of the important health recommendations [20]. In our study, the average physical activity was 24.42 minutes per day, the average consumption of fruits was 1.9 units per day and vegetables 2.1 units per day, and the amount of red meat consumption was 286.65 grams per week.

In the present study, only 1.1% were smokers, and among these people, the average smoking was about 15 cigarettes per day. In another report by the World Health Organization, the prevalence of daily smoking was 11.9% for the total population (20.9% for men and 2.9% for women) [21]. In the study of Savadpour et al., which was performed on 354 people over the age of 30 in Ardabil, 19.2% were smokers [14]. A 1998 study in Turkey found that 49.7% of men and 11.8% of women were daily smokers [22]. The rate of smokers in our study is lower than all the studies mentioned. High blood pressure is a major problem due to its association with coronary heart disease, cerebrovascular disease, and kidney disease [23].

In our study, 92.4% were in the normal range in terms of SBP and 95.1% in terms of DBP. The mean systolic and diastolic blood pressure of the subjects (including the mean of 3 systolic and diastolic blood pressures recorded in the last year for each person) was 108.14 and 68.26 mmHg, respectively. Based on systolic and diastolic blood pressure, respectively, 6.6% and 4.3% of the subjects were in the prehypertensive range, and 0.1% and 0.6% of the subjects were in the hypertensive group. The highest risk of cardiovascular disease is attributed to high blood pressure in the Iranian population [24].

This is because the prevalence of hypertension in the adult population over 18 years in Iran is between 17.3% and more than 20% [4] and about 26.9% in the population is between 25 and 64 years in Iran. [5] About 6.6 million Iranians between the ages of 25 and 64 had high blood pressure in 2005 [25]. According to a national study, among 69,722 Iranian adults aged 25 to 65, the prevalence of hypertension was 19.8% in men and 26.9% in women [26]. In a study of women over 30 years old in southern Iran, the prevalence of hypertension was reported to be 31.4% [27], and in the study of Gol et al., the prevalence of hypertensive patients based on SBP and DBP was 11.2% and 4.6%, respectively [10].

In a national study in Iran, the overall prevalence of systolic, diastolic, and also systolic or diastolic blood pressure was 4.2%, 5.4%, and 7.7%, respectively, with no significant difference in sex [28]. Various studies have reported a range of 17 to 50% control of hypertension in patients with hypertension treated in Iran based on different regions, age, and sex [3–5,24,25]. In the present study, the predominant type of HDL dyslipidemia was lower serum. High serum triglycerides, high cholesterol, and high serum LDL were other types of dyslipidemia in the subjects. In Latifi et al.'s study of 1,350 women over the age of 20 in Ahvaz, the prevalence of low HDL, hypertriglyceridemia, cholesterol, and high LDL was 54.7%, 65.1%, 47.5%, and 28.4%, respectively [29]. All listed values except the HDL level are higher than our results. However, in the study of Gol et al., abnormal levels of triglycerides, total cholesterol, LDL, and HDL were reported to be 32.5%, 25.7%, 17.8%, and 56.6% in the study population, respectively [10]. The results of our study are less. In the study of Khader et al., it was reported that in Jordanian women aged 25 to 85 years, serum triglyceride, cholesterol, and LDL levels were 38.9%, 51%, and 41.4%, respectively. However, the rate of people with low serum HDL (27.9%) is lower than our study [30].

The results of our study are even higher than those reported for 2601 women over the age of 20 in Turkey, with high triglyceride, cholesterol, and high LDL levels of 12.6%, 13%, 11.8%, and low HDL levels of 10.2%, respectively [31]. Comparison of age groups with risk factors showed that smoking, BMI, LDL, TG, cholesterol, FBS, and systolic and diastolic blood pressure should increase with age. Consumption of fruits and vegetables and the level of HDL also decrease with age. In the study of Gol et al., there was a very significant relationship between oxidized LDL (serum oxidized LDL) and increasing age [10]; and the relationship between LDL and age groups in our study is also significant. Obesity is associated with various metabolic disorders and increased cardiovascular mortality [32].

Comparison of BMI groups with risk factors showed that HDL, LDL, TG, FBS, and systolic and diastolic blood pressure should increase with increasing BMI. As mentioned in our study, BMI is significantly associated with aging. Similar to our findings, other studies have shown that obesity is less common in early adulthood, is associated with aging, and progresses more rapidly in women [33–35]. In the study of Mousavinasab et al. on 477 patients over the age of 35 who underwent angiography in Sari in 1994–95, the mean body mass index was 28.38. Of course, the average BMI in our study was 28.58; considering that in our study only women were included in the study and different studies have shown that in societies the average BMI in women is higher than men, we can say that the comparison of the difference between the means in the two studies is not significant, even though this value is higher in our study. In Sadeghi's commercial study, BMI was significantly associated with an increase in systolic and diastolic blood pressure, which was consistent with a study by the World Health Organization in Asian and African countries [36]. In his study, it was stated that the average body mass index in both sexes increases with age, and at least in the first three decades of life in adults, this increase occurs. But in women, it starts to decrease after the age of 54. Interestingly, in his study, physical activity was negatively associated with abdominal obesity and consumption of fruits and vegetables was not associated with abdominal obesity [8].

In our study, there is no strong relationship between fruit and vegetable consumption and body mass index. In the study of Savadpour et al., there was a strong significant relationship between blood pressure and high BMI and waist size [14]. Comparison of region and location with risk factors showed that consumption of fruits, vegetables, and red meat, HDL, LDL, TG, cholesterol, and systolic and diastolic blood pressure have a significant relationship with the location of people in Ardabil.

Comparison of education levels with risk factors showed that with increasing education, smoking and LDL, TG, cholesterol, FBS, and systolic and diastolic blood pressure decreased and HDL and consumption of red meat, vegetables, and fruits increased, indicating the effect of education on the level of knowledge and awareness of people and also the amount of their income. In our study, smoking was higher in single people than married people, and on the other hand, LDL, triglyceride, cholesterol, FBS, and systolic and diastolic blood pressure were higher in married people than in single people. Consumption of fruits, meat, and vegetables, HDL, and systolic and diastolic blood pressure were significantly higher among employed people than unemployed and housewives. In a review of the cardiovascular disease outlook over the past 40 years in Iran, the prevalence of cardiovascular risk factors including hypertension, diabetes mellitus, high LDL, low HDL, hypertriglyceridemia, hypercholesterolemia, obesity, and smoking among the population is currently 42.2%, 18.7%, 58.9%, 52.3%, 52.7%, 65.4%, 26.4%, and 13%, respectively [16]. The risk of cardiovascular disease associated with diabetes, hypertension, smoking history, abdominal obesity, and high LDL in Iranians was 9.9%, 36%, 5.5%, 18.9% and 24.1% [37]. Considering the prevalence of cardiovascular diseases and their risk factors, it has been concluded that the prevention and control of these modifiable risk factors can prevent up to 80% of cardiovascular diseases [14].

The models studied in our study show that the variables studied in our study predict 41.8% of changes in SBP and 39.6% of changes in DBP. In the study of Mousavinasab et al., in univariate logistic regression analysis, the variables of age, sex, smoking, fasting blood sugar, HDL, and triglyceride were significantly associated with increased vasoconstriction above 50%. This means that for every ten years of age, the risk of coronary artery disease is 58%, and men are 4.91 times more likely than women to have coronary artery disease. Smoking also increases the chance of coronary artery disease by more than 50%. HDL cholesterol below 40 for men and below 50 for women, fasting blood sugar above 110, and triglycerides above 150 also increase the chance of coronary artery disease by over 50%, 75%, and 61%, respectively.

In the multivariate logistic regression model, the odds ratio of sex, HDL, fasting blood sugar, triglyceride, and age increased and the smoking variable lost its significance [38]. Based on these results, it seems that facility-based opportunistic screening for cardiovascular risk factors is a viable option, and according to similar results in other studies, it is suggested that this is a regular practice in all PHCs in Ardabil. [15] Our findings provide evidence for health policymakers and other health officials about lifestyle problems in the study population. If an effective prevention strategy is not implemented, more disease burden is expected. Although short-term training programs have been shown to be effective in improving lifestyles, a sustainable education strategy and cost-saving policies supported by continuing media education and school-based education programs could be the starting point for a possible national program to control noncommunicable cardiovascular diseases [20, 39, 40].

### 4.1. Limitations

Because this study is retrospective, detailed information on how and what type of physical activity people do and the number of days of the week that people have effective and regular physical activity is not available. Another limitation of this study is the limitation of recording people's tests in electronic records (for example, items such as the levels of vitamins D, E, calcium, sodium, potassium, and alkaline phosphatase, which are important and effective factors associated with the disease. Are cardiovascular, not routinely measured and recorded in the population), and it is suggested that future research would examine the relationship between these and cardiovascular hazards.

### 4.2. Conclusion

According to the results obtained during the past years, the amount of smoking in the female population of Ardabil has been decreasing and the daily consumption of fruits in the diet of people has increased.

However, the rate of abdominal obesity (waist circumference above 88), obesity and overweight (high body mass index), high triglycerides, high cholesterol, and HDL and LDL outside the proper range are the most important and risk factors for cardiovascular disease in the women population of Ardabil.

## Figures and Tables

**Table 1:** Frequency distribution of the study's demographic variables.

Variable		*N*	Percent
Marital status	Single	30	3.0
	Married	967	96.1
Waist size (cm)	Normal (less than 80)	416	41.4
	High (equal to or above 88)	590	58.6
Education	Illiterate	66	6.6
	Primary	199	19.8
	Middle and high school	131	13
	Diploma	297	29.5
	Bachelor	274	27.2
	Masters	36	3.6
	PhD	3	0.3
Job	Housewife	829	82.4
	Governmental employee	138	13.7
	Freelance	39	3.9
Smoking	Yes	11	1.1
	No	995	98.9
Region	1	224	22.3
	2	208	20/7
	3	147	14.6
	4	205	20.4
	5	222	22.1
BMI	Low weight (less than 18.5)	1	0/1
	Normal (18.5–24.9)	221	22
	Overweight (25–29.9)	431	42.8
	Obese (above 30)	353	35.1

**Table 2:** Frequency distribution of clinical laboratory variables.

Variable		Number	%
Systolic blood pressure	Normal	930	92.4
	Pre-HTN	66	6.6
	HTN	10	1.0
Diastolic blood pressure	Normal	957	95.1
	Pre-HTN	43	4.3
	HTN	6	0.6
Triglyceride	Normal (less than 150)	439	43.6
	Borderline (150–199)	389	38.7
	High (200–499)	177	17.6
	Very high (above 500)	1	0.1
Cholesterol	Normal (less than 200)	682	67.8
	High (equal to or higher than 200)	324	32.2
HDL	Low (less than 50)	777	77.2
	Suitable (equal to or higher than 50)	229	22.8
LDL	Normal (less than 130)	861	85.6
	High (equal to or higher than 130)	145	14.4

**Table 3:** The mean and standard deviation of quantitative variables.

Variable	Mean	SD	The least	The most
Age (years)	42.98	8.45	30	60
Height (cm)	161.51	6.7	98	190
Weight (kg)	74.29	11.39	44	126
Waist circumference (cm)	91.32	10.4	55	125
Physical activity (daily activity rate) (minutes)	24.42	35.82	0	360
Fruits (units per day)	1.9	0.902	0	5
Vegetables (units per day)	2.1	1.07	0	8
Meat (grams per week)	286.65	174.64	0	1000
HDL (mg/dl)	43.6	10.84	15	95
LDL (mg/dl)	101.28	26.39	35	200
TG (mg/dl)	159.89	54.01	40	523
Cholesterol (mg/dl)	185.99	37.90	80	323
FBS (mg/dl)	94.62	13.38	63	190
Systolic blood pressure (mmHg)	108.14	10.23	85	147.67
Diastolic blood pressure (mmHg)	68.26	7.86	48.33	93.33
BMI (kg/m^2^)	28.58	5.09	17.15	46.80

**Table 4:** The relationship between clinical and laboratory cardiovascular risk factors and BMI.

Variable	BMI	Mean	SD	*P* value
HDL	Normal	46.88	11.17	<0.001
	Overweight	43.83	10.59	
	Obese	41.28	10.44	
LDL	Normal	91.46	23.72	<0.001
	Overweight	100.27	25.24	
	Obese	108.66	27.23	
TG	Normal	137.94	67/44	<0.001
	Overweight	155.38	36/47	
	Obese	179.23	29/60	
Cholesterol	Normal	175.68	32.47	<0.001
	Overweight	182.94	36.194	
	Obese	196.20	40.727	
FBS	Normal	90.91	19.692	<0.001
	Overweight	93.30	13.410	
	Obese	98.58	13.909	
Systolic pressure	Normal	101.93	8.432	<0.001
	Overweight	106.79	8.378	
	Obese	113.71	10.501	
Diastolic pressure	Normal	63.56	7.079	<0.001
	Overweight	67.57	7.064	
	Obese	72.08	7.409	

**Table 5:** The multiple linear regression model predicting SBP changes based on variables.

Model	*B*	Beta	*T*	*P* value
Constant	55.58		20.44	≤0.001
Age	0.349	0.289	10.621	≤0.001
Waist	0.178	0.182	5.163	≤0.001
Fruit	−1/521	−0.134	−5/111	≤0.001
Meat	0.006	0.104	4.001	≤0.001
LDL	0.052	0.134	4.741	≤0.001
Cholesterol	0.016	0.061	2.203	≤0.001
FBS	0.070	0.092	3.455	≤0.001
BMI	0.255	0.127	3.675	≤0.001
Cigarettes	0.693	0.117	4.781	≤0.001
	*R* ^2^: 0.424		Adj.*R*^2^: 0.418	

**Table 6:** The multiple linear regression model predicting DBP changes based on variables.

Model	*B*	Beta	*T*	*P* value
Constant	29.202		13.563	≤0.001
Age	0.227	0.244	8.84	≤0.001
Waist size	0.820	0.108	2.825	≤0.005
Fruit	−1.000	−0.115	−4.271	≤0.001
Meat	0.007	0.163	6.108	≤0.001
LDL	0.056	0.190	6.590	≤0.001
Cholesterol	0.013	0.61	2.149	≤0.032
FBS	0.058	0.099	3.623	≤0.001
Weight	0.115	0.166	4.506	≤0.001
Exercise	−0.018	−0.082	−3.202	≤0.001
	*R* ^2^: 0.401		Adj.*R*^2^: 0.396	

## Data Availability

The original data are available on request to the corresponding author, after the manuscript published. Whatever, we also considered to provide the original data in public repositories.

## Abbreviations

HTN: Hypertension
BMI: Body mass index
CVD: Cardiovascular disease
HDL: High-density lipoprotein
LDL: Low-density lipoprotein
TG: Triglycerides.
